# Mortality Table

**Published:** 1879-10

**Authors:** 


### MORTALITY TABLE.

Condensed from National Board of Health Bulletin for the four weeks ending August 30th, 1879.

| CITIES. | Estimated Population. | Deaths. | Death rate per 1000. |
|---|---|---|---|
| BALTIMORE, - - - - - | 400 000 | 596 | 19.39 |
| BOSTON, - - - - - | 365,000 | 667 | 23.45 |
| **BUFFALO,** - - - - | **170,000** | **218** | **15.39** |
| CHICAGO, - - - - - | 460,000 | 895 | 25.28 |
| CLEVELAND, - - - - - | 160,000 | 251 | 20.39 |
| LOUISVILLE, - - - - - | 175,000 | 180 | 13.27 |
| NEW YORK, - - - - - | 1,097,000 | 2188 | 25.01 |
| PHILADELPHIA, - - - - | 901,000 | 1345 | 19.07 |
| ROCHESTER, - - - - - | 90,000 | *61 | 17.62 |
| ST. LOUIS, - - - - - | 500,000 | 554 | 14.4 |

* Only two weeks reported by National Board of Health.

